# Essential oil from *Eugenia stipitata* McVaugh leaves exhibits antinociceptive effect via opioid receptor activation

**DOI:** 10.1007/s10787-026-02148-y

**Published:** 2026-03-02

**Authors:** Wêndeo Kennedy Costa, Priscilla Glazielly dos Santos de Moraes, João Guilherme Euzebio Souza, Paulo Henrique Andrade do Nascimento Silva, Hilary Araujo Dantas, Erica Kaylane da Silva, Laís Ruanita Leopoldina Galvão, Tamyres Suellen da Silva Freitas, Thaís Vitória Freitas de Souza, Alisson Macário de Oliveira, Márcia Vanusa da Silva, Maria Tereza dos Santos Correia

**Affiliations:** 1 Departamento de Bioquímica, Universidade Federal de Pernambuco, Recife, PE 50670-901 Brazil; 2 Programa de Pós-graduação em Ciências Farmacêuticas, Universidade Estadual da Paraíba, Campina Grande, PB 58429-500 Brazil

**Keywords:** Analgesia, Essential oils, Inflammatory pain, Opioid receptors, Pain

## Abstract

*Eugenia stipitata* McVaugh, a species of the Myrtaceae family, is widely distributed in tropical regions and has traditionally been used by local communities to treat mouth and throat inflammation, stomach pain, wounds, fever, diarrhea, and other inflammatory conditions. This study aimed to investigate the effects of the essential oil from *E. stipitata* (EsEO) in experimental pain models in mice, evaluating its potential as an alternative analgesic therapy. Mice were treated with EsEO and tested in formalin, tail immersion, and hot plate models to assess antinociceptive effects. Pharmacological antagonists were used to investigate the mechanism, suggesting opioid receptor involvement. Behavioral responses and latencies were measured post-treatment. EsEO significantly reduced neurogenic and inflammatory pain responses in all tests. In the formalin test, both early and late phases showed decreased pain behaviors. Similar reductions were observed in the tail immersion and hot plate tests. The major constituents appear to exert both central and peripheral analgesic effects. The use of antagonists indicated that the antinociceptive mechanism of the essential oil involves, at least in part, the modulation of the opioid pathway. EsEO exhibited significant antinociceptive activity and presents itself as a promising natural alternative for pain management. These findings support further research into the mechanisms and therapeutic potential of natural products for the development of safer and more effective analgesics.

## Introduction

Pain is an unpleasant sensory and emotional experience associated with actual or potential tissue damage and is one of the most frequently reported symptoms across various clinical conditions. Despite advances in the development of analgesics, many treatments still face significant limitations, such as undesirable side effects, tolerance, and dependence, underscoring the need for new therapeutic approaches. In this context, natural compounds have been widely explored as promising sources for the development of effective and safe antinociceptive agents (Farley et al. 2024; Rugnath et al. 2024; Soliman et al. 2024).

*Eugenia stipitata* McVaugh, a species belonging to the Myrtaceae family, is widely distributed in tropical regions and has traditionally been used by local communities for medicinal purposes. It is used to treat various conditions, such as inflammation of the mouth and throat, stomach pains, wounds, fever, diarrhea, pain, and other inflammatory conditions (Souza et al. 2018). Phytochemical studies have identified the presence of bioactive compounds, including essential oils, with potential therapeutic properties such as antimicrobial, antioxidant, and anti-inflammatory activities. However, there is a knowledge gap regarding the effects of this species, particularly in the context of pain modulation (Acosta-Vega et al. 2024; Dos Santos et al. 2023; Costa et al. 2022, 2024).

Essential oils, volatile compounds extracted from plants have attracted attention for their pharmacological activities, including antinociceptive effects demonstrated in various plant species. These properties are associated with the interaction of their constituents with specific molecular targets in the nervous system, such as opioid receptors, ion channels, and inflammatory mediators (Guedes et al. 2023; Lee and Hur 2022; Rivaz et al. 2021). Therefore, investigating the mechanism of action of *E. stipitata* essential oil may contribute to the development of novel analgesic strategies. This study aims to evaluate the antinociceptive mechanism of the essential oil extracted from *Eugenia stipitata* leaves, focusing on its effects on different types of pain, including inflammatory and neuropathic pain.

## Materials and methods

### Plant material and essential oil extraction

Leaves of *Eugenia stipitata* were collected in Exu, Pernambuco, Brazil (7° 21′ 16.3″ S 39° 53′ 15.5″ W). The plant material was registered in the National System for the Management of Genetic Heritage and Associated Traditional Knowledge (SisGen) under registration number A08E18B, and a voucher specimen (No. 13.054) was deposited at the Herbarium of the Regional University of Cariri. The leaves were subjected to hydrodistillation using a Clevenger-type apparatus for 4 h. The essential oil (EsEO) obtained was stored at 4 °C until pharmacological assays were performed. Hydrodistillation of *E. stipitata* leaves produced a clear yellow oil, with a yield of 6.65 g (0.73% w/w).

Gas chromatography analyses were conducted to determine the relative proportions of EsEO components using a Thermo TraceGC Ultra gas chromatograph equipped with a flame ionization detector (FID). All compounds, their retention indices, and relative proportions were previously reported, the majority of the compounds were identified with guaiol (13.77%), trans-caryophyllene (11.36%), β-eudesmol (8.13%), and γ-eudesmol (6.55%) (Costa et al. 2020).

### In vivo experiments

This study was approved by the Ethics Committee on Animal Use of the Federal University of Pernambuco (Approval No. 23076.011325/2018-77), and all procedures were conducted in accordance with Brazilian legislation on animal experimentation. Swiss mice were obtained from the Keizo Asami Immunopathology Laboratory (LIKA) animal facility. Animals weighed between 30 and 40 g, 8–10 weeks age. They were maintained under standard conditions (12-h light/dark cycle, 22 ± 2 °C, and 50–55% humidity) with free access to food and water. Male mice (*Mus musculus*, 30–35 g) were used in all experiments. The selection of males aimed to reduce hormonal variability associated with the estrous cycle, which can interfere with the nociceptive response and the assessment of analgesic activity. A total of 126 mice were used, distributed into groups of c animals each. The sample size was defined based on previous studies using similar experimental models and parameters, which indicated that this number is sufficient to detect statistically significant differences between groups, reducing animal overuse in accordance with the 3Rs principles.

### Formalin test

To assess pain behavior using the formalin test, animals were divided into six groups (n = 6) and treated with EsEO (40, 100, or 250 mg/kg, p.o.), saline solution (0.9%, p.o.), morphine (10 mg/kg, i.p.), or indomethacin (20 mg/kg, i.p.). Animals were then injected with 20 μL of 2.5% (v/v) formalin into the subplantar region of the right hind paw. The time spent licking the paw was recorded during the first 5 min (phase 1: neurogenic pain) and between 15 and 30 min post-injection (phase 2: inflammatory pain) (Hunskaar and Hole 1987).

### Tail immersion test

Animals were pre-screened 24 h before the experiment by immersing their tails in warm water (55 ± 1 °C). Those that kept their tails immersed for more than 5 s were excluded. The selected animals were divided into five groups (n = 6 per group) and treated with EsEO (40, 100, or 250 mg/kg, p.o.), saline (0.9%, p.o.), or morphine (10 mg/kg, i.p.). Tail immersion was performed at 0, 30, 60, 90, and 120 min after treatment. The latency to tail withdrawal was recorded, with a cut-off time of 20 s (Khatun et al. 2015).

### Hot plate test

Twenty-four hours prior to testing, mice were pre-screened using a hot plate apparatus (Insight, Brazil) maintained at 55 ± 0.5 °C. Animals that did not respond within 5 s were excluded. The selected animals were divided into five groups (n = 6 per group). On the test day, animals were treated with EsEO (40, 100, or 250 mg/kg, p.o.), saline (0.9%, p.o.), or morphine (10 mg/kg, i.p.), and then subjected to the hot plate test. The latency to discomfort reaction (paw withdrawal or jumping) was recorded, with a cut-off time of 20 s (Khatun et al. 2015).

### Investigation of the antinociceptive mechanism

To investigate the mechanism underlying the antinociceptive effect, animals were pretreated intraperitoneally with naloxone (2 mg/kg; non-selective opioid receptor antagonist), atropine (1 mg/kg; muscarinic receptor antagonist), glibenclamide (5 mg/kg; K^+^ channel blocker), prazosin (1 mg/kg; α₁-adrenergic antagonist), or yohimbine (1 mg/kg; α_2_-adrenergic antagonist). Animals were then treated with EsEO (250 mg/kg, p.o.) or morphine (10 mg/kg, i.p.) and subjected to the formalin test as described in “Formalin test” section.

### Statistical analysis

Data were analyzed using GraphPad Prism® version 8.0 and are expressed as mean ± standard deviation (SD). Statistical significance was determined using one-way analysis of variance (ANOVA), followed by Bonferroni or Dunnett’s post hoc tests, as appropriate. Differences were considered statistically significant at *p* < 0.001.

## Results and discussion

Previously, the EsEO was evaluated using the acetic acid-induced writhing test, and the results demonstrated significant antinociceptive activity. All tested doses (40, 100, and 250 mg/kg) reduced the number of abdominal writhes induced by acetic acid, with inhibition rates of 54.1%, 55%, and 56.6%, respectively, compared to the control group (*p* < 0.001). These findings indicate that EsEO has potential to attenuate acute visceral pain, as observed in this widely used experimental model (Costa et al. 2020).

In addition, it is important to note that the writhing test presents limitations in specificity for identifying antinociceptive compounds, as it involves multiple physiological pathways and may be influenced by nonspecific factors (Oliveira et al. 2018; Lima Araújo et al. 2024). Therefore, further studies were necessary to validate and elucidate the mechanisms of action of EsEO, particularly those involving specific pathways such as receptor participation, ion channels, or inflammatory mediators.

The formalin test is widely employed in pharmacological research to assess the antinociceptive activity of substances due to its ability to provide detailed insights into different types of pain and their underlying mechanisms. This model induces a biphasic nociceptive response: the first phase, known as the neurogenic phase, is characterized by direct activation of nociceptive sensory fibers, while the second phase, inflammatory in nature, is mediated by the release of inflammatory mediators such as prostaglandins, histamine, and bradykinin. This distinction allows for evaluation of whether the essential oil exerts analgesic effects through modulation of neurogenic, inflammatory, or both types of pain (Jimenez 2022; Nazari-Serenjeh et al. 2024; Wang et al. 2024).

**Fig. 1:**
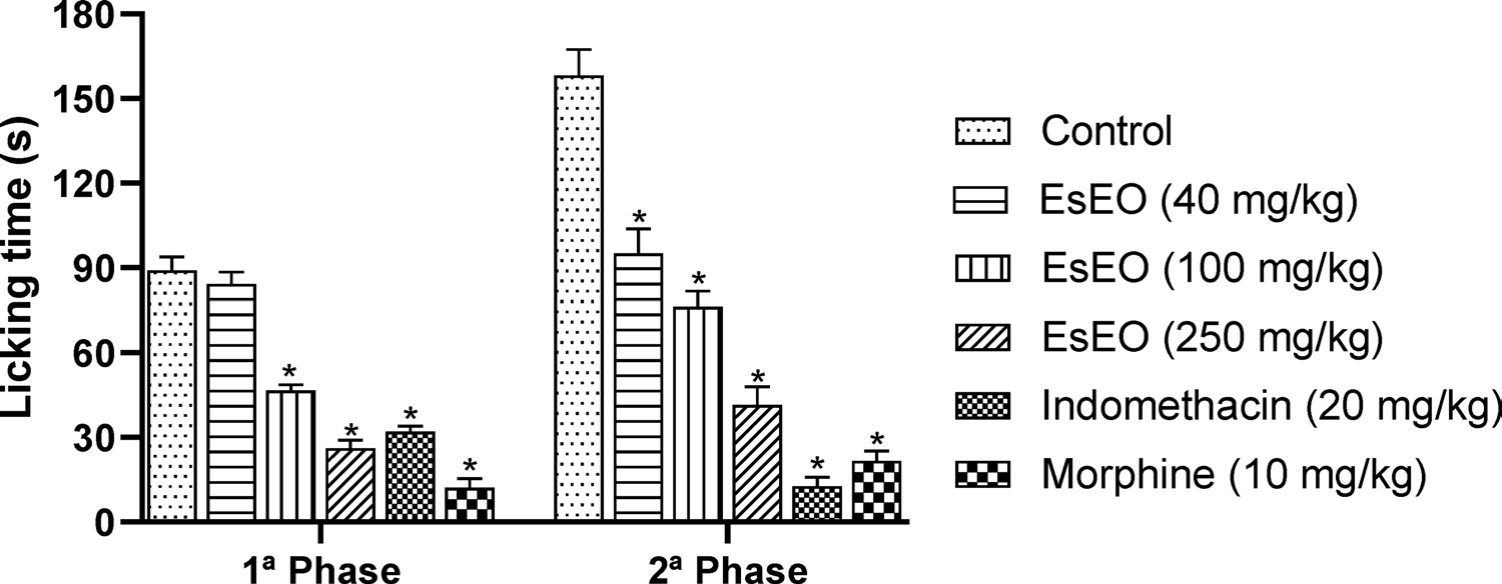
Effect of *Eugenia stipitata* essential oil (EsEO) on both phases of the formalin assay. *Eugenia stipitata* essential oil (EsEO). Values represent the mean ± SEM. *p < 0.001 compared with Control, one-way ANOVA followed by Dunnett’s Test

In the first phase of the formalin test, oral administration of EsEO (40, 100, and 250 mg/kg) significantly reduced the paw licking time compared to the control group by 5.44%, 47.87%, and 70.63%, respectively (Fig. 1). Indomethacin (20 mg/kg) and morphine (10 mg/kg), used as positive controls, significantly reduced the licking time by 64.04% and 86.51%, respectively. In the second phase, EsEO at 40, 100, and 250 mg/kg produced marked reductions of 39.93%, 51.76%, and 73.75%, respectively. The positive controls indomethacin and morphine also showed substantial inhibition, reducing the licking time by 91.99% and 86.30%, respectively, when compared to the control.

The compounds guaiol, trans-caryophyllene, β-eudesmol, and γ-eudesmol, present in the essential oil of *E. stipitata* (Costa et al. 2020), play a key role in modulating pain in the experimental formalin test model. Trans-caryophyllene, a sesquiterpene known as a selective agonist of cannabinoid type 2 (CB2) receptors, likely contributes significantly to pain reduction, particularly during the inflammatory phase. Its interaction with CB2 receptors may modulate the release of inflammatory mediators such as cytokines and prostaglandins, thereby attenuating secondary neuronal sensitization. Additionally, its activity on ion channels may also exert modulatory effects during the neurogenic phase (Giordano 2005; Andrade-Silva et al. 2016; Koyama et al. 2019; Hashiesh et al. 2021; He et al. 2021).

Guaiol, β-eudesmol, and γ-eudesmol also exhibit well-documented anti-inflammatory and antinociceptive properties, which are relevant to both phases of the test. During the neurogenic phase, these compounds may reduce nociceptor excitability through the modulation of channels such as Transient Receptor Potential Vanilloid 1 (TRPV1) and Transient Receptor Potential Ankyrin 1 (TRPA1), which are directly activated by the chemical stimulus of formalin. In the inflammatory phase, they may inhibit the release of pro-inflammatory mediators such as TNF-α, IL-1β, and prostaglandins, thereby reducing amplification of the pain response (Seo et al. 2011; Moon et al. 2018; Liktor-Busa et al. 2021; Tharabenjasin et al. 2024).

These findings support the notion that the antinociceptive effect observed for EsEO in the formalin test is mediated, at least in part, by the interaction of these compounds with pathways involved in pain and inflammation. Furthermore, the potential synergy between these sesquiterpenes may enhance the overall efficacy of the essential oil. Based on this, additional studies targeting specific mechanisms were conducted to confirm these interactions (Déciga-Campos et al. 2021; Ridouh and Hackshaw 2022).

The tail immersion and hot plate tests are widely used to evaluate the antinociceptive activity of compounds, providing valuable information regarding the involvement of centrally mediated mechanisms. Both models assess reflex responses to thermal nociceptive stimuli but differ in key aspects that allow for a more detailed characterization of analgesic potential (Le Bars et al. 2001; Khatun et al. 2015; Oliveira et al. 2018).

**Fig. 2:**
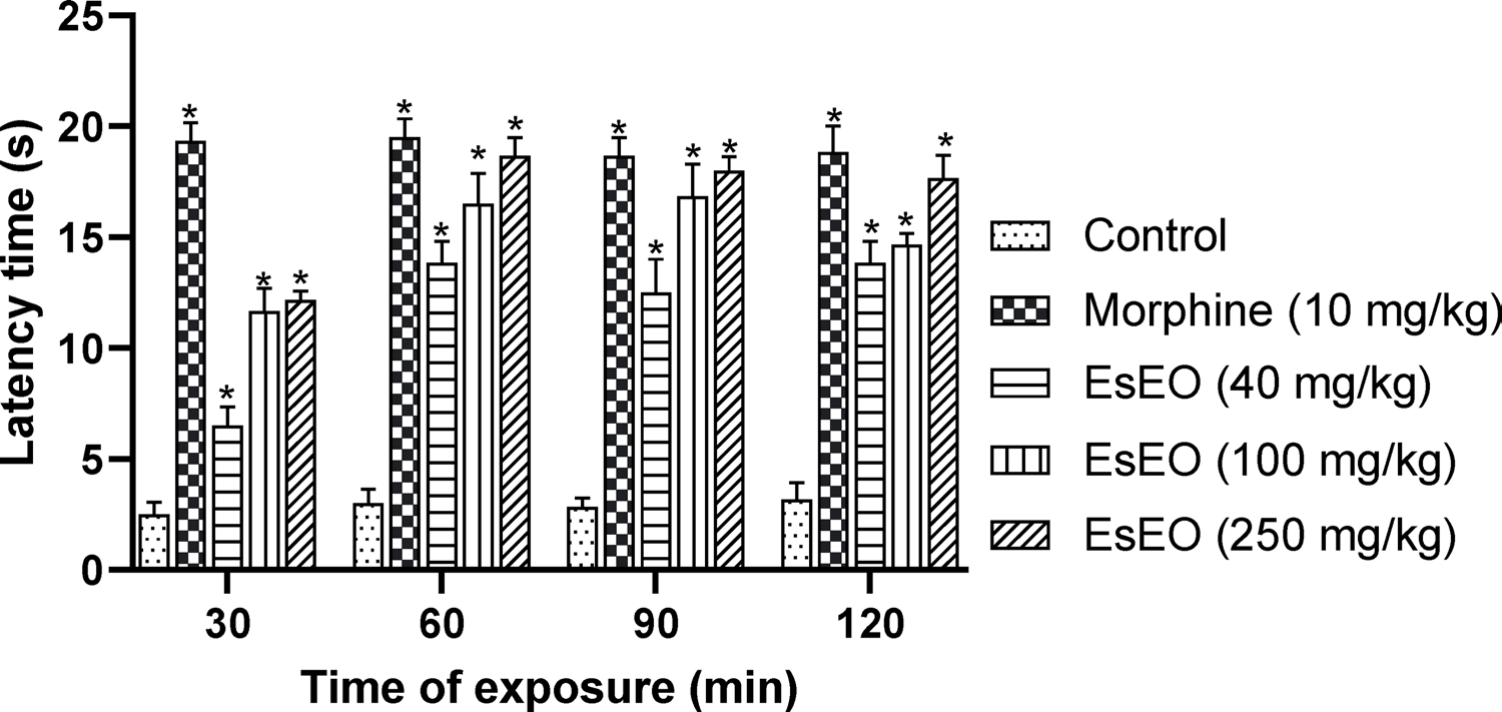
Effect of *Eugenia stipitata* Essential Oil (EsEO) on tail-immersion assay. *Eugenia stipitata* essential oil (EsEO). Values represent the mean ± SEM. *p < 0.001 compared with Control, one-way ANOVA followed by Dunnett’s Test

In the tail immersion test, the latency time of animals treated with EsEO and morphine increased significantly (*p* < 0.001) compared to the control group 30 min after treatment (Fig. 2). At the 60-min time point, the group treated with EsEO at 250 mg/kg showed maximal latency response.

In the tail immersion assay, the latency to tail withdrawal in response to a thermal stimulus is used as an indicator of analgesia. This model primarily reflects activity in spinal and supraspinal nociceptive pathways and is highly sensitive to analgesics that act on opioid receptors, particularly μ-opioid receptor agonists (Khatun et al. 2015; Oliveira et al. 2018; Costa et al. 2024).

**Fig. 3:**
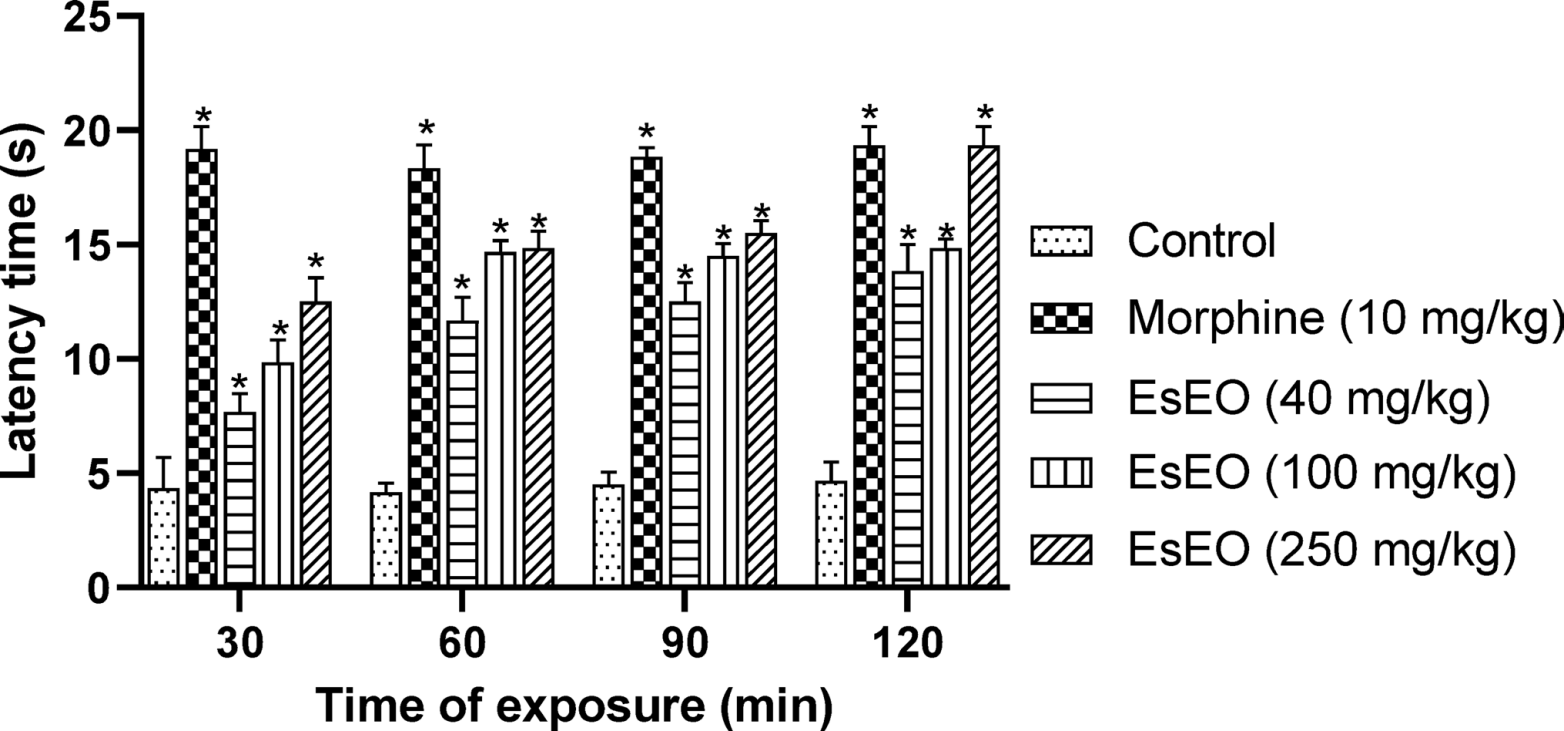
Effect of *Eugenia stipitata* essential oil (EsEO) on the hot-plate. Values represent the mean ± SEM. *p < 0.001 compared with Control, one-way ANOVA followed by Dunnett’s Test

In addition, the hot plate test was conducted. The results demonstrated that treatment with EsEO at doses of 40, 100, and 250 mg/kg significantly prolonged the latency to response on the hot plate compared to the control group, particularly at 30 min post-administration. In contrast, the group treated with morphine exhibited an increase in latency time as early as 30 min after administration, reaching maximal efficacy (Fig. 3).

The hot plate test involves measuring the latency to display reflex behaviors such as paw licking or jumping in response to a thermal stimulus. This model is useful for evaluating pain modulation mechanisms mediated by supraspinal pathways and involves the participation of various receptor classes, including opioid receptors and TRPV1 channels (Le Bars et al. 2001; Costa et al. 2024).

The significant increase in response latency observed in both tests suggests that EsEO acts on central nociceptive pathways, possibly mediated by opioid receptors or the modulation of ion channels involved in pain perception. This finding is consistent with the presence of bioactive compounds in the essential oil, such as trans-caryophyllene, guaiol, and eudesmols, which have well-documented antinociceptive properties (Hashiesh et al. 2021; He et al. 2021; Liktor-Busa et al. 2021; Tharabenjasin et al. 2024).

Trans-caryophyllene may modulate sodium and calcium channels, reducing neuronal excitability, and there is evidence that it can potentiate the analgesic effects of opioids, suggesting an indirect interaction with these receptors (Hashiesh et al. 2021). Guaiol primarily acts by modulating ion channels, which play a crucial role in the transduction of nociceptive stimuli (Dutra et al. 2016; Souza et al. 2024). Eudesmols (α and β), in turn, exert their effects through the suppression of pro-inflammatory cytokines and oxidative stress, both of which contribute to pain sensitization (Moon et al. 2018).

Confirming the involvement of specific receptors requires additional studies, such as the use of selective antagonists, to precisely identify the underlying mechanisms. Therefore, to assess the potential mechanism of EsEO’s antinociceptive action, animals were pretreated with specific antagonists, followed by administration of EsEO at 250 mg/kg and subsequent evaluation in the formalin test.

**Fig. 4:**
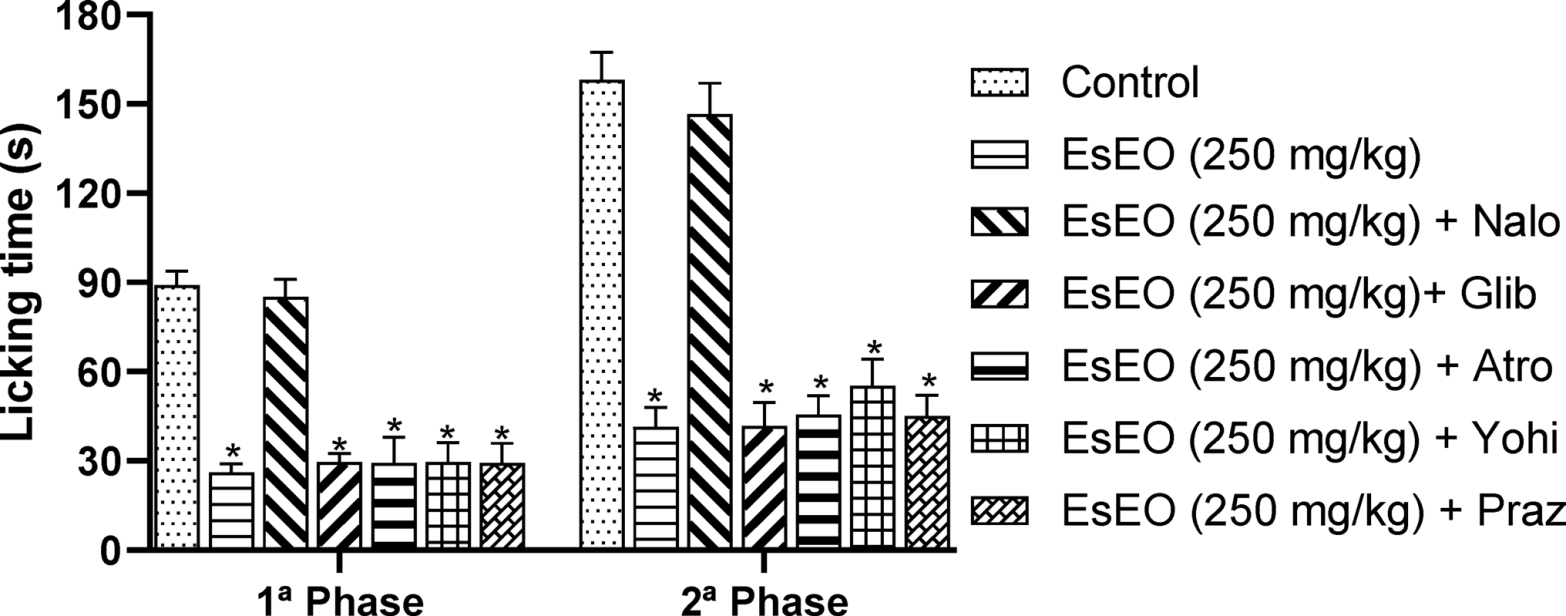
Investigation of mechanisms of antinociceptive activity of the *Eugenia stipitata* essential oil (EsEO) in the formalin test. Values represent the mean ± SEM. *p < 0.001 compared with Control, one-way ANOVA followed by Dunnett’s Test

The results presented in Fig. 4 show that pretreatment with naloxone (an opioid receptor antagonist) completely reversed the antinociceptive effect of EsEO, reducing the activity in the first phase to 4.31% and in the second phase to 7.27%. These findings suggest that the antinociceptive action of EsEO is mediated, at least in part, by the opioid system. In contrast, pretreatment with atropine (a muscarinic receptor antagonist), glibenclamide (a K⁺ channel blocker), prazosin (an α₁-adrenergic antagonist), or yohimbine (an α₂-adrenergic antagonist), followed by treatment with EsEO (250 mg/kg), did not produce significant changes compared to the group treated with EsEO alone.

The antinociceptive activity of EsEO through opioid receptors is of great relevance, particularly as it represents a natural and promising alternative for pain management. Activation of μ, δ, and κ opioid receptors plays a crucial role in pain modulation, providing potent analgesia by reducing the transmission of pain signals in both the central and peripheral nervous systems. This action may offer effective relief for various types of pain, including chronic and neuropathic pain, which often show resistance to conventional treatments (Król et al. 2024; Staedtler et al. 2024).

Furthermore, the use of natural compounds, such as those found in the essential oil of *Eucalyptus stipitata*, may reduce the risks associated with synthetic opioids, such as dependence, tolerance, and respiratory depression, making it a safer and more sustainable option. The essential oil also possesses complementary properties, such as anti-inflammatory and antioxidant effects, which help reduce inflammation and oxidative stress, factors that contribute to pain sensitization and perpetuation (Javed et al. 2021; Corasaniti et al. 2023; Sattayakhom et al. 2023).

During the trials, no signs of toxicity, behavioral changes, or mortality were observed at any of the tested doses of the essential oil, suggesting a safety profile compatible with experimental use. These findings are consistent with previous studies that reported no significant toxic effects in mice treated with Eugenia stipitata essential oil (Costa et al. 2020). Therefore, the essential oil can be considered safe under the experimental conditions employed.

## Conclusion

The results of this study demonstrated that EsEO exhibits significant antinociceptive activity, with notable effects in reducing acute visceral, neurogenic, and inflammatory pain. Furthermore, the increased latency time in the tail immersion and hot plate models suggests an action on central nociceptive pathways, mediated by opioid receptors. The study reinforces the relevance of EsEO as a promising natural alternative for pain management, with analgesic, anti-inflammatory, and antioxidant potential.

## Data Availability

No datasets were generated or analysed during the current study.
